# Parenting styles and the mental health of left-behind children in China

**DOI:** 10.3389/fpsyg.2025.1332977

**Published:** 2025-10-10

**Authors:** Lingrui Wu, Di Qi

**Affiliations:** Department of Sociology, School of Public Administration, Hohai University, Nanjing, China

**Keywords:** left-behind children, mental health, CEPS, response exploration, mental resilience

## Abstract

The issue of left-behind children is a prominent social challenge that has surfaced in recent years as China’s modernization process advances. Due to national policies and economic limitations, most migrant parents opt to leave their children behind in their hometowns and entrusted to other relatives. The prolonged absence of parents, inadequate caretaker ability of other relatives, and limited resources in communal living conditions lead to mental development problems in left-behind children to varying degrees. Such children are more vulnerable to depression, anxiety and loneliness compared to their peers. Thus, the mental health issues of left-behind children ought to receive requisite attention. This paper utilizes the 2013–2014 survey data from the China Education Panel Study (CEPS) for an empirical research aiming to analyze quantitatively the effect of parenting styles on mental health of left-behind children, along with an examination of the mediating role of mental resilience, the results of which are as follows: first, positive parenting behavior significantly reduces mental health problems among left-behind children, whereas positive parenting attitudes do not have a significant effect; second, mental resilience mediates the influence of parenting behavior on children’s mental health but not the effect of parenting attitudes; and third, both positive parenting behavior and parenting attitudes significantly enhance the mental resilience of left-behind children.

## 1 Introduction

The problem of left-behind children in China has emerged in the 1980s and gained significant attention in society since 2004 (Duan and Zhou, 2005). Its essence lies in the separation of parents and their underage children caused by population mobility (Pan and Ye, 2009). The absence of parents has negative consequences on children’s physical and mental development, social interactions as well as mental well-being (Zhang et al., 2014). Thus, the governance of this problem has become a crucial issue that the Party and the State must address urgently. The laws and regulations outlined in Opinions of the State Council on Strengthening the Care and Protection of Rural Left-behind Children (2013) by the Ministry of Education, the National Plan for the Development of Children in (Liu, 2014) by the Circular of the General Office of the State Council on the issuance of the national plan for the development of children in impoverished areas (2014), and the The State Council issued the Opinions on Strengthening (2016) promote the support and protection of left-behind children from a global perspective. These laws and regulations are characterized by flexibility, development and sustainability, and they offer strong operability solutions. Nonetheless, a recent meta-analysis revealed that left-behind children have a higher detection rate for depressive symptoms (around 29%) as well as for social anxiety (36.1%) when compared to their non-left-behind peers (Liu et al., 2021). This suggests that addressing the issue of left-behind children is a challenge and protracted process.

The mental health of children left behind is currently a key topic of widespread academic concern. Mental health refers to an intact state of cognitive rationality, emotional stability, appropriate behavior, and harmonious interpersonal relationships formed in the process of individual development, and is an important component of health. Some studies have indicated that the mental health of left-behind children is normal and not significantly different from that of their non-left-behind peers (Yue et al., 2006; Hu et al., 2007). However, other scholars hold different views, contending that the phenomenon of children left behind has a negative impact on their mental health and personality traits. In addition, mental issues among children left behind in junior high schools are particularly acute (Gao, 2008) evident in lower levels of self-consciousness, greater loneliness and higher levels of social anxiety. Notably, the fact that their parents work outside of the home is a significant risk factor for the mental health of these children (Zhao et al., 2012).

Given the urgency of resolving the issue of left-behind children, it is highly significant to study their mental well-being. Currently, the research on left-behind children’s mental health issues has two important deficiencies. Firstly, research primarily focuses on children’s mental health status, emotions and interpersonal relationships, with little investigation being conducted on the influence of parenting styles, behaviors and attitudes on the mental health of left-behind children. Secondly, Secondly, the role played by children’s mental resilience in the mental health of children left behind and its heterogeneous characteristics have hardly been fully explored by the academia. Considering the aforementioned shortcomings, this paper hopes to propose the influence of parenting styles on left-behind children’s mental health by taking family parenting styles as the entry point, introducing the mediating variable of mental resilience, and analyzing the microdata through field research and utilizing the data from the 2013–2014 annual survey of the China Education Panel Study (CEPS), and exploring the role played by mental resilience in the mental health of left-behind children. Finally, policy recommendations and intervention measures to promote the mental health of left-behind children are proposed.

## 2 Literature review and hypothesis

### 2.1 Parenting styles and mental health

Parenting styles refer to a range of behaviors and attitudes in parenting and values towards child development (Darling and Steinberg, 1993). The reason why parenting styles are subdivided into parenting behaviors and parenting attitudes in this paper is that parenting attitudes refer to parental evaluations of parenting pressures, and parenting behaviors refer mainly to parental feeding behaviors and early education behaviors toward children. These two have distinctive features and can well reflect the parenting styles. The family is a crucial environment for individual growth, parenting styles and behaviors have far-reaching impacts on children. As a result, there have been extensive discussions on parenting styles in the academia. Baumrind (1971), a renowned American psychologist, initially introduced the idea of family parenting. Through his research, he divided parenting styles into three categories: authoritative, authoritarian, and tolerant. Later, Maccoby and Martin further categorized parenting styles based on the dimensions of responsiveness and demandingness. They identified four types: authoritative type with high responsiveness and high demandingness, authoritarian type with low responsiveness and high demandingness, permissive type with high responsiveness and low demandingness, and neglectful type with low responsiveness and demandingness. Some researchers also believe that parenting style is complex and comprises various perspectives and aspects. Therefore, a single dimension cannot comprehensively summarize parenting behavior. There is a large number of well-established measurement scales on parenting styles at home and abroad. For instance, Perris developed *the Evaluation of Parenting Behavior (EMBU)*, which is the most influential and widely used questionnaire abroad. Arrindell narrowed down the dimensions to trust and encouragement, authoritarianism, spoiling, emotional warmth and neglect, and developed *the Simplified Parenting Behavior Scale (s-EMBU)*. A widely used parenting style scale in China is Yue Dongmei’s Family Parenting Style Scale, which incorporates Perris’s EMBU scale within the Chinese context. The scale eliminates entries from the EMBU scale and categorizes the parenting styles of fathers and mothers into six and five dimensions, respectively. Based on this, Wang (1998)divided the EMBU questionnaire into positive dimensions of parenting style (emotional warmth, understanding) and negative dimensions of parenting style (punishment and harshness, denial, over-interference, and protection). *The Chinese version of the Simplified Parenting Questionnaire (s-EMBU-C)* contains 21 questions and retains the three core dimensions of the original English version, namely denial, emotional warmth, and overprotection.

Research into the impact of parenting styles on the mental health of left-behind children has two main perspectives. The first perspective is to compare the parenting styles of left-behind with non-left-behind children and analyze the differences in their mental health; the second perspective analyzes the impact of different types of guardianship on the mental health of left-behind children from the perspective of guardianship type. A questionnaire survey was conducted by Liu and Chen (2018) among primary and secondary school students in Yunnan Province. The survey found that, in comparison non-left-behind children, left-behind children experienced less warm parenting styles. Allowing mothers to act as caregivers, it can reduce the problematic behaviors of left-behind children. However, it may create tensions in the mother–child relationship; According to the study by Huang and Li (2012), the effects of various parenting styles on various left-behind children are inconsistent. Overall, adopting positive and warm parenting styles rather than refuse to discipline them or spoil them is more beneficial to the healthy growth of left-behind children. Grandparenting (also known as inter-generational parenting) is one of the forms of guardianship that has been extensively researched. Considering reality and relevant literature, it is easy to conclude that grandparents tend to spoil their grandchildren through their parenting styles. Excessive care and protection significantly predict emotional and behavioral problems in children (Liu, 2003). As Zhu (2015) research findings, children who are raised solely by grandparents are more likely to have emotional, personality, and interpersonal problems, as well as worse social adjustment and lower levels of mental health compared to children raised in other parenting styles. This is because although grandparents are able to take care of their grandchildren physically, they cannot replace their parents emotionally. Deficiencies in emotions would impact the development of grandchildren’s secure attachment and social skills.

In general, positive parenting attitudes and behaviors facilitate high levels of self-esteem, optimism, self-control, social and moral maturity, while negative parenting styles such as verbal aggression, corporal punishment, and mental detachment can impede children’s development. For instance, Li et al. (2000) demonstrated that negative parenting styles are associated with a higher probability of kids experiencing negative emotions such as anxiety and depression.

### 2.2 The mediating factor: mental resilience of left-behind children

Mental resilience, also known as mental toughness, is a significant component of mental resources. It describes the process through which individuals encounter either a stressful situation or continued stress and their protective resources interact with the stressor to reduce or eliminate any negative consequences (Rutter, 2012).

1. On the one hand, according to Liu and Chen (2018), left-behind children’s mental resilience is generally at a middle to upper level, and there are differences in the mental resilience level of left-behind children under different parenting styles, which are as follows: ① Parents with high resilience tend to provide them with more love, care and encouragement. In contrast, parents with low resilience may be colder, more rejecting and overprotective; ② Parental care and encouragement of autonomy are significantly associated with positive mental resilience of left-behind children. Conversely, indifference, rejection and overprotection have a significantly negative association with such resilience; ③ Parental care and rejection can significantly and positively affect the mental resilience of left-behind children, which is a protective factor. However, father’s indifference and rejection and mother’s overprotection both negatively impact such resilience, making them risk factors; ④ Parenting styles can affect the mental resilience of left-behind children to varying degrees, with mother’s parenting style having a greater influence than the father’s (Liu and Jin, 2018). This conclusion aligns with Tang (2012), Li et al. (2016), and Zhu et al. (2019) findings, which suggest that parenting style and caregivers’ mental resilience have a predictive effect on left-behind children’s mental health. At present, domestic academics give insufficient attention to the relationship between parenting styles and the mental resilience of left-behind children. Parental departure causes a significant change in family structure for left-behind children during their childhood, which can have a major impact on their lives. Hence, it is important for the state and society to focus on the influence of parenting styles on the mental resilience and mental health of left-behind children.
2. On the other hand, mental resilience has an impact on the mental health of children left behind. Numerous studies have confirmed that the mental health of left-behind children generally experience some mental health issues. Nevertheless, individual mental resilience levels account for the variance in mental health status among left-behind children. Currently, domestic research on the mental resilience levels of left-behind children primarily examines their perspective of positive psychology. Song et al. (2013) explored the mental resilience levels of left-behind children from this perspective and confirmed a significant positive correlation between the mental resilience levels and mental health of the children, with higher levels of mental resilience corresponding to higher levels of mental health. Zhou et al. (2013) demonstrated that mental resilience plays a vital role in mental health protection, serving as a significant moderator between life events and mental health by effectively mitigating the negative impact of unfavorable circumstances. Furthermore, mental resilience exhibits a significant modulating effect between social support and mental health. Moreover, it can strengthen the protective effect of social support during low life events. In various studies, other researchers have emphasized the importance of mental resilience in different ways——suggesting that it positively affects children facing adversity, and that negative environments or experiences do not inevitably impact the individual negatively. Instead, the protective factors from the individual, the family, and external sources work together in countering the negative environmental effects. Additionally, one’s coping processes with stress are vital for overcoming adverse situations. It is possible to divide protective factors into individual, family, and extra-familial ones. Gender, life satisfaction, self-efficacy, personality traits, and subjective well-being are some examples of individual factors. Parent labor status, parenting styles, parent–child contact and attachment, number of children in the family, and family socio-economic status are some examples of family factors. Social support, peer relationships at school, and teacher-student relationships are categorized as external factors.

## 3 Empirical methods

Built on the previous research, this study was designed to investigate the relationship between different parenting styles and mental health, and thus proposes the hypothesis H1: There are notable discrepancies in the mental health of left-behind children under different parenting styles. On a more subdivided dimension, good parenting behaviors (Hypothesis 1a) and parenting attitudes (Hypothesis 1b) can have a positive impact on the mental health of children left behind. In order to test the aforementioned hypothesis and to empirically estimate the relationship, this article initially employed the multivariate regression method, with the model specification presented here (Equation 1).


(1) $$Y_{i}=\alpha +\beta *X_{i}+\gamma *Z_{i}+\mu_{s}+\lambda_{t}+\varepsilon \;$$

where $Y_{i}$ denotes the dependent variable, i.e., the mental health of child *i*, $X_{i}$ indicates the independent variable, i.e., the different parenting styles *i*, and β represents the coefficient of interest that interprets their causal relationship. In addition, this model controls for a vector of demographic characteristics of individuals, households, and external factors, $Z_{i}$ . Furthermore, $\mu_{s}\;$ and $\lambda_{t}$ are the fixed effects of province and year, respectively.

After the first step, we want to explore the mechanism behind this relationship. The influence of parenting styles may not be straightly linked to children’s mental health. Thus, some mediational factors may play roles in the connection between both ends of the hypothetical relationship. Consequently, we put forth the second set of hypotheses:

Hypothesis 2a: Positive parenting behaviors have a positive effect on left-behind children’s mental resilience.

Hypothesis 2b: Positive parenting attitudes have a positive effect on left-behind children’s mental resilience.

Although the importance of children’s mental resilience has been paid attention to, and the significant effects of parenting styles on left-behind children’s mental resilience as well as the effects of mental resilience on left-behind children’s mental health have also been discussed respectively, how much of a role it plays in the mechanism of parenting styles’ effects on left-behind children’s mental health and what its heterogeneous characteristics will be still need to be further explored. Considering these two points, we use structural equation modeling to explore mediating effects.

Hypothesis 3: Mental resilience plays a mediating effect in parenting styles on the mental health of left-behind children.

Hypothesis 3a: Mental resilience plays a mediating effect in parenting behaviors on the mental health of left-behind children.

Hypothesis 3b: Mental resilience mediates the effect of parenting attitudes on the mental health of children left behind.

## 4 Data and variables

This study uses data from the China Education Panel Survey (CEPS) baseline database that was collected by the National Survey Research Center at Renmin University of China (NSRC) during the autumn and spring semesters of 2013 and 2014. The database primarily employs questionnaires and multi-stage probability proportional to size (PPS) sampling methods to survey students, their parents, classroom teachers, and school leaders. The purpose is to examine how family, school, and social factors influence individuals and their education, which enhances the reliability and accuracy of empirical analysis.

Table 1 displays the variables used in the paper and corresponding descriptive statistics. The mental health is considered the dependent variable. The question reads as follows: ‘Have you experienced any of the following feelings in the last 7 days?’ The question further divides the available options into five subcomponents: ‘frustration’, ‘depression’, ‘unhappiness’, ‘life is not fun’, and ‘sadness’. The possible responses for each question option range from ‘never’ to ‘always’. The measurement of the five sub-questions is deemed effective, as demonstrated by a Cronbach’s alpha coefficient of 0.856, a KMO coefficient of 0.86, and a Bartlett’s significance of 0.00.

**Table 1:** Regression results of parenting attitudes on children’s mental health under left-behind conditions.

	(1)	(2)	(3)	(4)	(5)
**Variables**	**Frustration**	**Depression**	**Unhappiness**	**Life is not meaning**	**Sadness**
(homework and exams) Do not care	0.168*	0.303***		0.420***	0.420***
	(−0.0874)	(−0.0963)		(−0.0988)	(−0.0988)
Very strict			−0.284***		
			(−0.0938)		
Care but not strict	0.0842**	0.0784**	−0.179**	0.140***	0.140***
	(−0.034)	(−0.0374)	(−0.0895)	(−0.0384)	(−0.0384)
(Daily home time) Care but not strict	−0.0745**	−0.0834	−0.0829	−0.097	−0.097
	(−0.0341)	(−0.0622)	(−0.0607)	(−0.0641)	(−0.0641)
(Choice of friends) Care but not strictly	−0.0763**	−0.0462	−0.130***	−0.0655	−0.0655
	(−0.0366)	(−0.0401)	(−0.0447)	(−0.0413)	(−0.0413)
Very strict			−0.0949*		
			(−0.0518)		
(Dressing) Care but not strict	−0.0623*	−0.016		0.0289	0.0289
	(−0.037)	(−0.0455)	(−0.0443)	(−0.0419)	(−0.0419)
(Time for Internet) Very strict	0.0158	−0.032	−0.103	−0.0749*	−0.0749*
	(−0.065)	(−0.0423)	(−0.0696)	(−0.0435)	(−0.0435)
Do not care		0.041		0.148**	0.148**
	(−0.0727)		(−0.0748)	(−0.0748)	
(Time for television) Care but not strict	−0.0247	−0.0341	−0.00376	−0.0734*	−0.0734*
	(−0.0523)	(−0.039)	(−0.056)	(−0.0401)	(−0.0401)
(Performance in the school) Very strict			−0.117*		
			(−0.0691)		
Constant	2.402***	2.232***	2.954***	1.886***	1.886***
	−0.0817	−0.111	−0.103	−0.0936	−0.0936
Observation	4,270	4,252	4,260	4,245	4,245
*R*-squared	0.011	0.008	0.015	0.023	0.023

Standard errors in parentheses. ***p < 0.01, **p < 0.05, *p < 0.1. Bold values are used to highlight regression results with significant levels, and bold is used only for emphasis.

The independent variables of this study are mainly parenting styles, which include two main categories: parenting behaviors and parenting attitudes.

### 4.1 Parenting attitudes

The variable related to parenting attitudes in the CEPS 2014 questionnaire is question B23. The stem of the question is, “Do your parents discipline you strictly in the following things?” The questions were set to “homework and exams,” “performance at school,” “going to school every day,” “what time you get home every day,” “who you make friends with,” “how you dress,” “how much time you spend on the internet,” and “how much time you spend watching TV.” The answers to these questions included three categories: “do not care,” “care but not strict,” and “very strict.” In the actual measurement, the Cronbach’s alpha coefficient of the parenting attitude was 0.78, the KMO coefficient was at 0.80, and the Bartlett’s significance was 0.00.

### 4.2 Parenting behaviors

The questions in the CEPS 2014 questionnaire that deal with parenting behaviors mainly cover six areas, B22 (parents’ supervision of children’s studies), B24 (parents’ discussion of problems with children), B25 (children’s relationship with parents), and B28 (frequency of children’s interactions with parents). Parental supervision of children’s studies was assessed by the frequency with which parents checked children’s homework and tutored children’s homework during the week. The answers to the questions were “never,” “1 or 2 days a week,” “3 or 4 days a week,” and “almost every day.” The questions in the questionnaire on the relationship between the child and his/her parents were used to determine the closeness of the child to his/her father or mother by direct questioning. The options were “not close,” “average,” and “very close.” Variables related to parent–child interactions in the questionnaire included the following: children discussing issues with their parents (what happens at school, children’s relationships with friends, children’s relationships with teachers, children’s moods, and children’s worries or concerns), and the frequency of doing the following things with their parents (including eating, reading, watching TV, doing sports, visiting, and going out). The options for how often children discuss issues with their parents are “never,” “sometimes,” and “often.” The frequency of children’s participation in activities with their parents was “never,” “once a year,” “once every 6 months,” “once a month “, “once a week” and “more than once a week.” The KMO value of the parent–child interaction is 0.78–0.84, and the KMO coefficient varies between 0.71 and 0.79, while the Bartlett’s significance is 0.00, indicating that it is suitable for factor analysis.

Question A12 from the CEPS 2014 survey provides a good assessment of children’s mental resilience. Question A12 reads, “Recalling your time in Grade 6, do you agree with the following descriptions of yourself?” The question comprises seven subcomponents with specific options, which are, “Even if I was a little unwell, I still try to attend school,” “I try my best to do my homework even if it’s something I do not like,” “I keep trying my best to do my homework even if it takes a long time,” “I can express my opinions clearly,” “I have quick reflexes,” “I can learn new things quickly” and “I am curious about new things,” The responses include for options: “Totally disagree,” “Do not quite agree,” “Comparatively agree, “and “Totally agree” 4 options.

This study controlled for other potential factors that may affect mental health in children. Student-specific characteristics were analyzed, including the type of hukou (0 = non-agricultural hukou, 1 = agricultural hukou), the child’s gender (0 = female, 1 = male); whether or not the child was an only child (which was transformed into a dummy variable in this study, with 0 = No and 1 = Yes). Parental characteristic variables included: family economic conditions (which were categorized in this study as difficult [very difficult and relatively difficult], medium [relatively wealthy] and wealthy [very wealthy] with values 0 = difficult, 1 = medium, 2 = wealthy); and parental education level was based on a nine-category in the CEPS 2014 survey and was converted to years of education and included as a continuous variable in the model by Tu et al. (2017). To be Specific: 0 = no education; 6 = elementary school; 9 = junior high school; 11 = secondary/technical school; 12 = vocational high school/high school; 15 = university college; 16 = undergraduate college; 19 = postgraduate student and above; parental occupation (this study refers to the type of class division in China and divides the parental occupation into a four-categorical variable, which is specifically as follows: white-collar workers [including state organizational institution leaders and staff, middle and senior managers of enterprises/companies], blue-collar workers [including skilled workers and general employees], farmers/autonomous [including farmers and self-employed] and others [including jobless, unemployed, laid-off and others]. Four categorical variables were assigned the following values: 0 = White-collar workers, 1 = Blue-collar workers, 2 = Farmers/autonomous; 3 = Other).

## 5 Results

To empirically test the hypothesis that whether parenting styles may positively impact on children’s mental health, Tables 1, 2 displays the stepwise regression results. Since parenting styles consists of two different dimensions in this paper, Tables 1, 2 represents on each dimension, respectively.

**Table 2:** Regression results of parenting behavior on children’s mental health under left-behind conditions.

	(6)	(7)	(8)	(9)	(10)
Variables	Frustration	Depression	Unhappiness	Life is not fun	Sadness
(Relationship with mother) Not get along well		0.205***	0.122		0.226***
		(−0.0789)	(−0.0777)		(−0.0783)
Relationships in general	−0.146**			−0.222***	
	(−0.0738)			(−0.0827)	
Close relationship	−0.260***	−0.165***	−0.153***	−0.441***	−0.0765*
	(−0.0723)	(−0.0419)	(−0.0413)	(−0.081)	(−0.0416)
(Relationship with father) Not get along well		0.214***		0.169**	
		(−0.0641)		(−0.0672)	
Relationships in general	−0.149**		−0.196***		−0.149**
	(−0.0599)		(−0.0632)		(−0.0636)
Close relationship	−0.218***	−0.116***	−0.279***	−0.119***	−0.231***
	(−0.0607)	(−0.0394)	(−0.064)	(−0.0413)	(−0.0643)
(Frequency of exercise with parents) Never	0.0607	−0.0593	0.151**	0.0115	0.1
	(−0.0667)	(−0.0646)	(−0.0703)	(−0.0675)	(−0.0709)
Once every 6 months		−0.143*		−0.0717	
		(−0.0845)		(−0.0883)	
Once a week	0.0343	−0.109	0.0022	−0.139*	−0.0191
	(−0.0769)	(−0.0712)	(−0.081)	(−0.0744)	(−0.0817)
	(−0.166)	(−0.178)	(−0.175)	(−0.186)	(−0.177)
(Frequency of dinner with parents) Never				0.227*	
				(−0.126)	
Once per year	0.00183	−0.202*	−0.118	0.00146	−0.401***
	(−0.109)	(−0.118)	(−0.115)	(−0.0971)	(−0.116)
Once every 6 months	0.03	−0.11	−0.102		−0.209*
	(−0.112)	(−0.121)	(−0.119)		(−0.119)
Once a month	−0.0377	−0.0374	−0.0356	0.0441	−0.305**
	(−0.114)	(−0.123)	(−0.12)	(−0.104)	(−0.121)
Once a week	0.0437	−0.0543	−0.0445	0.0653	−0.319***
	(−0.105)	(−0.113)	(−0.111)	(−0.0975)	(−0.112)
More than once a week	−0.0367	−0.121	−0.0912	0.0977	−0.394***
	(−0.0956)	(−0.103)	(−0.101)	(−0.0851)	(−0.102)
(Frequency of reading with parents) Once per year	0.0059	0.169*	0.0225	0.103	0.103
	(−0.085)	(−0.0895)	(−0.0898)	(−0.0957)	(−0.0905)
Parents never instructed homework.	−0.0212	0.0507	0.0967**	0.160***	0.0173
	(−0.0527)	(−0.0565)	(−0.0477)	(−0.059)	(−0.048)
Constant	2.686***	2.276***	2.501***	2.253***	2.609***
	−0.19	−0.184	−0.192	−0.196	−0.192
Observations	4,002	3,985	3,990	3,982	3,988
*R*-squared	0.039	0.063	0.07	0.066	0.044

Standard errors in parentheses. ***p < 0.01, **p < 0.05, *p < 0.1. Bold values are used to highlight regression results with significant levels, and bold is used only for emphasis.

First of all, when considering only the effect of parenting attitudes on the mental health of left-behind children, the sample is split, i.e., Models 1–5 are each of the five dimensions of children’s mental health. Controlling for variables such as children’s individual characteristics and family characteristics, parenting attitudes all have some degree of influence on children’s mental health.

Specifically, Model 1 in Table 1 shows that when adopting “do not care” or “care but not strict” attitudes towards children’s exams and studies, it will exacerbate the “depression” aspect of their mental health. In contrast, when parents adopt “care but not strict” attitude towards their children’s daily home time, choice of friends and dressing, it reduces the incidence of mental health problems (depression) in children. For instance, the incidence of mental ill-health in children is, respectively, −0.0745, −0.0763, and −0.0623 times higher than the incidence of mental health with a significant impact on the above three indicators. The occurrence of mental health issues related to frustration, depression, life is not fun, and sadness in Models 2, 4, and 5 was more probable when parents adopted “do not care” or “care but not strict” attitudes towards their children’s education and examinations. It is noteworthy that parenting styles with very strict attitudes did not have a pronounced influence on children’s mental health. This is because left-behind children, who represent a special group, lack care and attention from their parents or relatives in their daily lives. If parents are unable to timely attend to children’s studies, these events may lead to feeling undervalued, which can consequently result in varying psychological issues. Conversely, receiving assistance and attention from relatives can improve a child’s subjective well-being and to some degree, lessen their mental issues. During adolescence, children are in a unique developmental stage. It is advisable for parents to avoid displaying authoritarian tendencies to prevent any counterproductive outcomes that could lead to rebelliousness. Simultaneously, peer groups play a significant role in a child’s growth and development. When faced with problems, teenagers often turn to their peers for solace and solutions, rather than their parents, guardians, or teachers. Peer groups are typically composed of individuals with similar age, social status, interests, and family background. They provide emotional support and empathy to children in need. In particular, for children left behind, who cannot express their negative emotions freely to their guardians, peers can provide emotional comfort and academic support.

Furthermore, as evident from Models 4 and 5, when parents exert strict control over their children’s Internet usage, the incidence ratio of children’s mental ill-health is −0.0749 times higher than the ratio for mental health. Conversely, when parents display the “do not care” behavior towards their child, the incidence of mental ill-health amongst children is 0.148 times higher than the incidence for mental health. The prolonged absence of parents may increase the risk of antisocial behavior among left-behind children. In today’s rapid technological advancements and internet proliferation, children have more avenues to access the Internet. Due to their lack of cognitive development and exposure to different perspectives, children are incapable of performing a balanced evaluation of information. Given the abundance of mixed and unreliable content available on the Internet, parental supervision of a child’s online activity becomes paramount. Without proper guidance, it is easy for children to be swayed by misinformation, posing a grave risk to their mental health.

For the analysis of parenting behaviors’ effects on the mental health of left-behind children, the sample was divided. Scenarios of mental health distress, namely frustration, depression, unhappiness, life is not fun, and sadness are represented by Models 6–10 in this study.

Children’s closeness to their parents has a significant impact on their mental health. From models 6–10, it can be seen that when children’s closeness to their father is shown to be “not close,” it will aggravate children’s mental problems in the three aspects of depression and life is not fun. When the relationship between the child and the father is “very close,” it will weaken all aspects of the child’s mental problems, and the ratios of frustration, depression, unhappiness, life is not fun, and sadness are −0.218, −0.116, −0.279, −0.119, and −0.231, respectively. Even if a child’s closeness to his or her father is only “average,” it reduces the emergence of emotions such as unhappiness, frustration and sadness. Secondly, when left-behind children’s closeness to their mothers is very low, it aggravates the children’s mental problems of depression and sadness; children with average and high closeness to their mothers have a significant negative effect on their unhealthy mental problems, especially when the children are very close to their mothers, their incidence of mental unhealthy ratios are −0.260, −0.165, −0.153, −0.441 times higher than the ratio of the incidence of mental health ratios, all of which have a significant effect. This indicates that the higher the level of closeness with parents, the higher the level of mental health of children. In reality, the proportion of fathers going out to work is high, most of the left-behind children lack father’s love and father’s care during their growing up process, and they long for father’s attention and support. Increased intimacy with fathers can contribute to children’s self-happiness. More understanding and support will reduce the incidence of their mental problems such as depression and unhappiness. Compared to fathers, mothers tend to play a warm role in the family, and most children have more frequent and deeper communication with their mothers, as well as receiving more encouragement and care from their mothers. This is conducive to reducing children’s mental health problems of anxiety and depression, whereas the lack of in-depth communication with their mothers or lack of care from their mothers is likely to cause their mental health problems.

The effect of family discipline on children’s mental health is reflected in this table in the frequency of parental guidance on children’s homework. Models 6, 7, and 10 demonstrate that when parents provide 1–2 days per week, 3–4 days per week, or almost daily instructions to their children on doing homework, it has no significant effect on their mental health. In contrast, in models 8 and 9, when parents never instruct their children on doing homework during the week, the likelihood of their children experiencing mental health issues, such as unhappiness and meaninglessness in life, increased. As demonstrated by the incidence ratios of mental health, which were 0.0967 and 0.160 times higher than the incidence ratios of mental ill-health, children were more likely to experience mental health issues when their parents were lax in disciplining them regarding their homework. These findings are consistent with the conclusions drawn from the study on the effect of parenting attitudes on the mental health of left-behind children. Activities between parents and children have a significant impact on the mental health of children. For instance, if we take the frequency of the variable ‘having dinner with parents’, children who never have dinner with their parents display a 0.227 times higher frequency of mental ill-health as compared to their mental well-being. Focused model 10 showed that when children have dinner with their parents once a year, half a year, once a month, once a week, or once a week (especially when the frequency was once a week and more than once a week), the incidence of children’s “sadness” mental problems was significantly reduced. Specifically, the incidence of mental ill-health in children is −0.401, −0.209, −0.305, −0.319 and − 0.394 times higher than the incidence of mental health. Therefore, parents having dinner with their children frequently can reduce the likelihood of mental problems and increase their level of mental health.

As discussed above, in order to reveal the impact mechanism on parenting styles on children’s mental health, and test the hypotheses of mediation effect, this paper uses the structural equation model to estimate the direct and indirect influences. Results indicate that the model showed a good overall fit on the general dimensions (RMSEA = 0.033, GFI = 0.987, AGFI = 0.980, NFI = 0.981, IFI=CFI = 0.982, RMR = 0.030), and good parenting styles had a significant negative effect on mental health problems (*β* = −0.151, *p* < 0.001). This implies that improved parenting styles result in a lower occurrence of children’s mental health problems.

**Figure 1:**
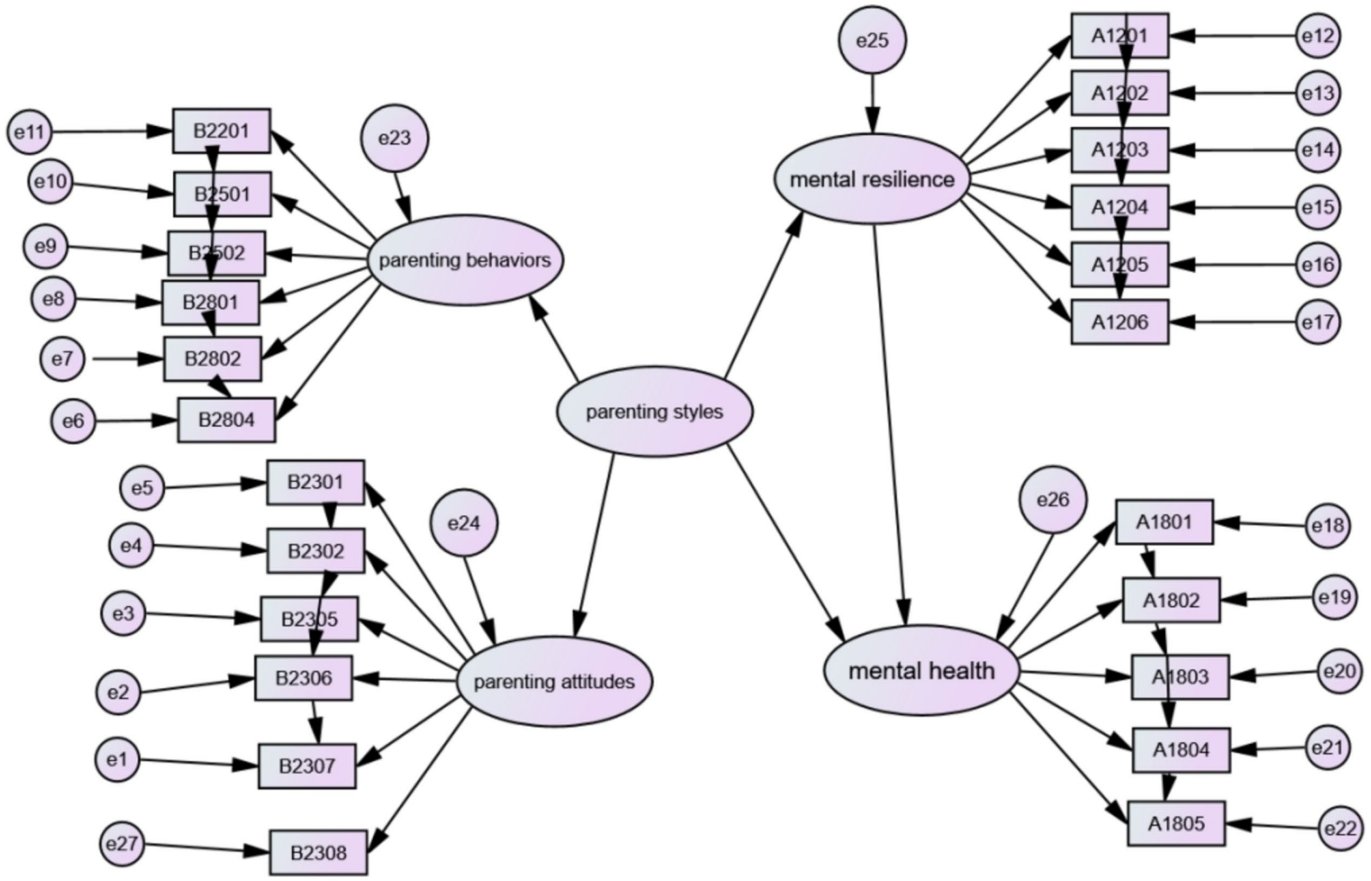
A model of the mediating role of mental resilience in the relationship between left-behind children’s mental health and parenting styles (overall dimension).

Figure 1 shows the path diagram of the indirect effect of parenting styles on children’s mental health problems mediated by mental resilience. The results show that the model fits well overall (RMSEA = 0.030, GFI = 0.983, AGFI = 0.977, NFI = 0.976, IFI=CFI = 0.977, RMR = 0.034); the direct effect of parenting styles on mental health is significant (*β* = −0.148, *p* < 0.001), and at the same time, the parenting styles → mental resilience (*β* = 0.154, *p* < 0.001) as well as the path of mental resilience → mental health (*β* = −0.304, *p* < 0.001) were significant. Mental resilience played a mediating effect in the mechanism of the effect of parenting styles on mental health, with a mediating effect of 0.154 × −0.304 = −0.021, and the direct effect of parenting styles on mental health was significant (*β* = −0.148, *p* < 0.001), so mental resilience played a partially mediating effect in the mechanism of the effect of parenting styles on mental health, with a partially mediating effect accounting for the total effect of the ratio is −0.021/(−0.021 + −0.148) = 12.43%, so the hypothesis “there is a mediating effect of mental resilience in the mechanism of the influence of parenting styles on children’s mental health” is supported. The study tested the theoretical hypotheses that parenting style has a significant positive impact on mental resilience, and that mental resilience significantly affects mental health negatively. The study also proposed that mental resilience acts as a mediating factor in the relationship between parenting styles and mental health, and that the mediating effect drives the pathway of “parenting styles → mental resilience →mental health.” This mediating effect represents the theoretical driving mechanism linking the three variables.

**Figure 2:**
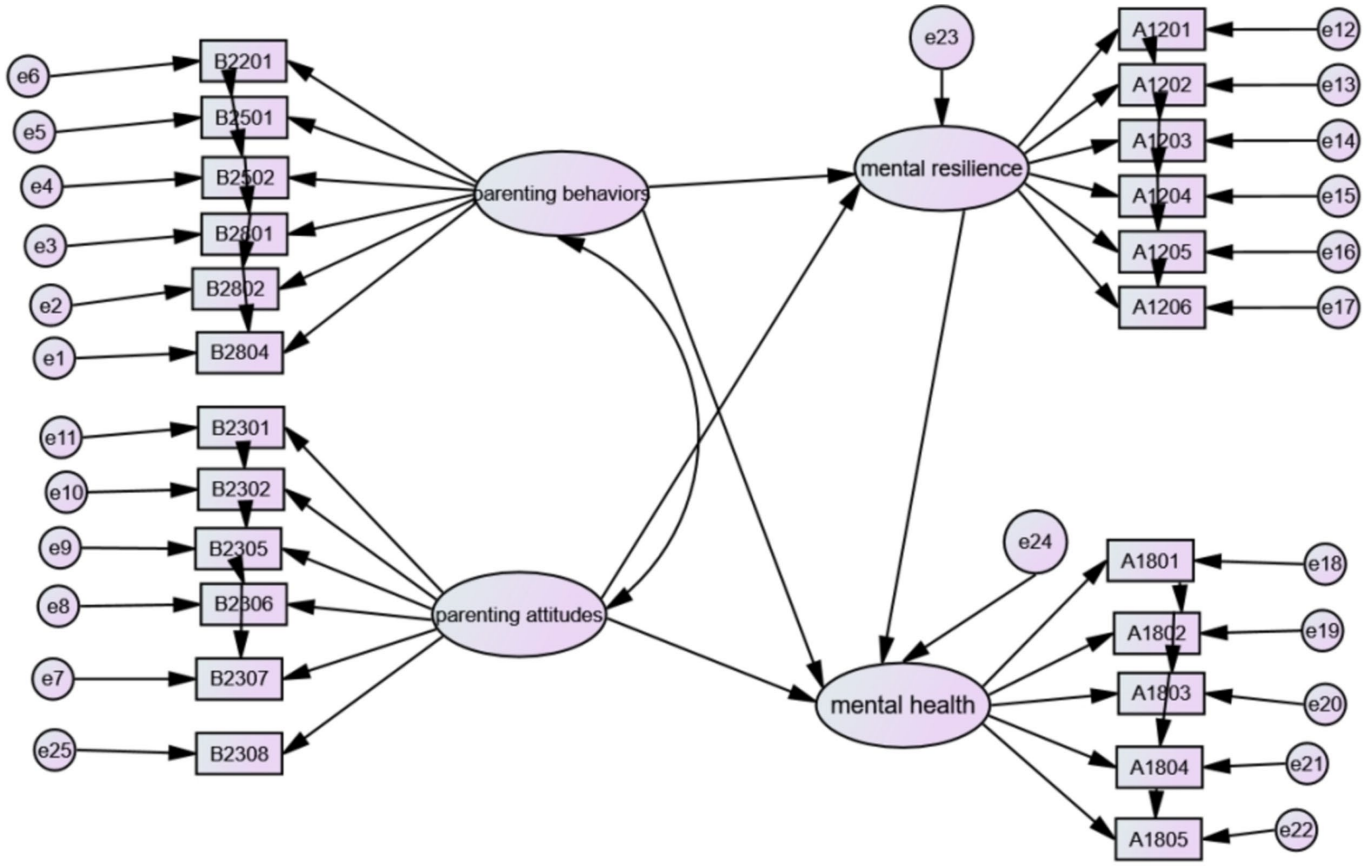
A model of the mediating role of mental resilience in the relationship between left-behind children’s mental health and parenting styles (breakdown of dimensions).

Based on the above research content and objective, along with the scale content, a diagram (sub-dimension) was created to represent the indirect influence of parenting styles on children’s mental health mediated by mental resilience, as depicted in Figure 2.

We estimated the parameters of the model using the maximum likelihood estimation method and found that the model was well-fitted. After revising the correlation between independent variables, all fitting indicators reached the fitting standard (RMSEA = 0.034, GFI = 0.979, AGFI = 0.968, NFI = 0.968, IFI = 0.969, CFI = 0.969, RMR = 0.045). We can observe the significance of the path coefficients of mental resilience-mediated parenting on the mental health of left-behind children from Table 3.

**Table 3:** Paths diagram.

Paths	Standardized path factor	*p*-value
Parenting behaviors → mental resilience	0.108	***
Parenting attitudes → mental resilience	0.049	***
Mental resilience → mental health	−0.335	***
Parenting behaviors → mental health	−0.147	***
Parenting attitudes → mental health	0.016	0.04

The level of significance is indicated by a *p*-value, with ***indicating *p* < 0.01, i.e., extremely significant difference.

From the above table of path coefficients and their significance, it can be seen:

### 5.1 Parenting behaviors → mental resilience path analysis

Based on the standardized path coefficients and their significance tables presented above, it can be observed that the coefficient of the impact of parenting behaviors in parenting styles on mental resilience has reached a significant level: parenting behaviors → mental resilience (*β* = 0.108, *p* < 0.001). Therefore, the hypothesis stating that “parenting behaviors in parenting styles have a positive and significant influence on mental resilience” is therefore supported.

### 5.2 Parenting attitudes → mental resilience path analysis

The standardized path coefficients and their significance tables reveal that the coefficient for the relationship between parenting attitudes within parenting styles and mental resilience has reached a significant level: parenting attitudes → mental resilience (*β* = 0.049, *p* < 0.001). Therefore, it can be concluded that the hypothesis that “parenting attitudes in parenting styles have a positive and significant influence on mental resilience” is therefore supported.

### 5.3 Mental resilience → mental health path analysis

The above standardized path coefficients and significance tables show that there is a significant impact of mental resilience on children’s mental health: mental resilience → mental health (*β* = −0.335, *p* < 0.001). Therefore, we can support the hypothesis that “good mental resilience significantly affects mental health problems” is therefore supported.

### 5.4 Analysis of the mediating effect of mental resilience in the pathway of parenting behavior on mental health

The analysis shows that parenting behaviors have a positive relationship with mental resilience (*β* = 0.108, *p* < 0.001), and mental resilience has a negative relationship with mental health (*β* = −0.335, *p* < 0.001), indicating that mental resilience partially mediates the relationship between parenting behaviors and mental health, and the mediating effect coefficient is −0.036 (0.108 × −0.335 = −0.036). The partial mediating effect of mental resilience accounts for 20.0% (−0.036/(−0.036–0.147) = 20.0%) of the total effect of parenting behaviors on children’s mental health. The direct effect of parenting behaviors on children’s mental health is statistically significant, hence, mental resilience plays a significant partial mediating role in the effect of parenting behavior on children’s mental health. Consequently, the hypothesis that mental resilience plays a mediating role in the relationship between parenting behaviors and children’s mental health is supported.

### 5.5 Analysis of the mediating effect of mental resilience in the pathway of parenting attitudes on mental health

From parenting attitudes → mental toughness (*β* = 0.049, *p* < 0.001), mental toughness → mental health (*β* = −0.335, *p* < 0.001), it can be seen that mental resilience plays a mediating role in the effect of parenting behaviors on mental health, the mediating effect is 0.049 × −0.335 = −0.016, the ratio of the mediating effect to the total effect is −0.016/(−) 0.016 + 0.016 = 0, so mental resilience does not play a mediating role in the effect of parenting attitudes on children’s mental health. Therefore, the hypothesis “there is a mediating effect of mental resilience in the mechanism of the influence of parenting attitudes on children’s mental health” was rejected.

## 6 Conclusion and discussion

### 6.1 Conclusion

This study utilized the 2013–2014 CEPS dataset and used structural equation modelling to test for mediating effects. In summary, the findings show how family parenting styles affect children’s mental health and which factors play a mediating role.

First, although studies have been conducted to discuss the link between parenting styles and children’s mental health, the specific mechanisms of influence are not clear, and few studies have explored the effects of mental resilience on mental health using it as a mediating variable. The empirical results suggest that positive parenting behaviors in parenting styles can positively and significantly affect left-behind children’s mental health, while parenting attitudes do not have a significant effect. Compared with non-left-behind children, left-behind children feel less warm parenting styles. Therefore, in terms of parenting behaviors, some studies point out that guardians adopting positive and warm parenting styles rather than refusing to punish and spoiling and protecting parenting styles are more conducive to the healthy growth of left-behind children. For example, the relationship and frequency of interaction with parents embodied in the model belongs to a positive family therapy approach, which may be conducive to the resolution of left-behind children’s mental health problems by setting boundaries between parents and children, adjusting family outcomes and other techniques.

Second, the findings suggest that both good parenting behaviors and parenting attitudes contribute to the psychological resilience of left-behind children. More researchers believe that psychological resilience is positive for children in adversity, and that adverse environments or experiences do not necessarily have a negative impact on individuals. The key to successful stress coping lies in whether an individual has protective resources to cope with stress, such as positive cognition, good parent–child relationships, and parenting styles. For left-behind children, the change in family structure due to parental absence has caused them to experience greater life changes in childhood. Li et al.’s (2016) study showed that parents’ emotional care for their children is one of the most important factors affecting left-behind children’s psychological resilience, which in turn leads to the differentiation of left-behind children in terms of their mental health level. The findings of this study also partially confirm the third hypothesis of the article, which is that mental resilience plays a mediating role in the influence of parenting behavior on children’s mental health, while there is no mediating effect in the influence of parenting attitude on children’s mental health. In future research, the academic mechanisms of the mental resilience -parenting behavior-mental health transmission effect can be explored in depth.

### 6.2 Discussion

The family is often the first place where children socialize and learn to interact with others. Parents, being the primary caregiver of children, have the responsibility to provide a conducive environment for their healthy physical and mental growth through proper education. A relaxed and pleasant family environment, along with parental accompaniment and scientific parenting techniques, plays a vital role in the growth and development of children. Despite unavoidable difficulties faced by migrant parents, the education and mental health of their children cannot be overlooked. Parents and other guardians should make joint efforts to create a warm and harmonious family atmosphere and a friendly environment for left-behind children. This will prevent them from feeling lonely and losing their sense of identity and belonging to the family. This study focuses on the core aspects of parenting styles at the micro level, aiming to provide feasible interventions that can help improve the mental health of left-behind children.

#### 6.2.1 Developing scientific parenting concepts

Parents and other guardians engage in self-learning, self-education and self-awareness while raising their children. Establishing effective parenting concepts necessitates parents and temporary guardians to supplement their education methods according to the child’s behavior and needs at different developmental stages; Additionally, they should correct their educational attitudes, continuously learn new educational concepts, and cultivate a sense of responsibility. Moreover, focus should be given to the academic challenges faced by left-behind school-age children and help foster their interest and curiosity in learning. Migrant parents can maintain close contact with schools and teachers to monitor their children’s academic progress. Thirdly, creating a stable, supportive and comforting family environment is crucial for left-behind children. Similar to kinship parenting, uncles, aunts, or other relatives often act as the guardians of left-behind children. Children who are left behind and living with other families may experience a sense of alienation. Consequently, some of them may become cautious in their lives, and the more sensitive children may develop low self-esteem and other emotional issues (Qi, 2022). Therefore, it is vital to establish a democratic and equal family atmosphere to address this situation. Parents should learn to respect their children’s choices instead of indulging them or neglecting them. Fourthly, it is crucial to assist left-behind children in developing positive self-esteem and self-confidence. Parents should encourage their children to try new things and believe in their abilities. Fifth, cultivating positive behaviors and values is necessary. Left-behind children should be guided by parents or temporary guardians to develop positive behaviors and values through leading by example. In addition, left-behind children should be encouraged to participate in social activities and groups, while being trained in appropriate social skills.

Apart from parents, grandparents are the primary caregivers. Grandparents are often constrained by traditional family education concepts and a lack of cultural knowledge, leading to conservative attitudes and beliefs about the education and care of left-behind children. Studies have shown that children in intergenerational parenting have poorer mental health, experiencing more anxiety, sensitivity and loneliness, are disadvantaged in interpersonal communication (Gao, 2010), and are prone to issues in cognitive, emotional and social development (Li et al., 2021). However, the responsibility of intergenerational caregiving and the situation of being left behind can negatively impact the mental well-being of grandparents. Such negative emotional states could indirectly affect the mental health of left-behind children (Qu et al., 2019). Therefore, fathers have a responsibility to provide love and care to their parents, enabling them to maintain positive emotional states that, in turn, enable them to offer positive emotional support and love to left-behind children.

#### 6.2.2 Explore correct parenting styles

The frequency of parent–child conflicts under authoritative, democratic, and permissive parenting styles has been analyzed by some scholars. It has been found that the frequency of parent–child conflicts is lower in the democratic parenting style (He, 2008). As far as parenting styles are concerned, authoritarian parenting styles have a higher likelihood of leading to frequent parent–child conflicts and parent–child conflicts when compared to authoritative parenting styles (Smetana, 1995). According to overseas studies, the authoritative parenting style, characterized by “warmth and care + guidance and control + moderate or higher educational expectations for behavior and achievement,” is considered to be the most effective parenting style (Rohner and Rohner, 1981). This paper observes that the incidence of mental health problems among left-behind children is lower when their guardians show a “very strict” attitude towards them instead of a “not strict” or “do not care” attitude. Parents and other guardians ought not to treat their children in a simple, rough, or scolding manner. While interacting with their children, parents should strive to comprehend their children’s perspective, adapt their thinking accordingly, and effectively communicate their expectations. Moreover, they should discuss critical events that left-behind children may experience during their growth process and make decisions together by listening to their opinions. During significant events in a left-behind child’s developmental process, parents should discuss them with their child, listen to their opinions and make decisions collaboratively. Despite facing limitations of time and space, migrant parents need to increase interaction and communication with their children whenever possible, to actively participate in their Childrens’ growth process and avoid being absent.

Some studies suggest that the absence of education on parent–child relationships for left-behind children can make them more susceptible to negative emotions and cause mental health issues as they lack education and family support (Fan and Sang, 2005). Furthermore, ineffective parent–child education and lack of supervision of left-behind children may increase their vulnerability to safety hazards, poor academic performance, and mental struggles (Tang and Lu, 2006). Therefore, it is evident that parents and temporary guardians receiving proper training on effective parenting is the best solution to lessen physical and mental health problems of left-behind children. One possible strategy is to utilize village (neighborhood) committees to connect with pertinent educational resources while bringing in experts in the field of pedagogy to deliver recorded lectures (Sanders, 2012). Furthermore, a resource-sharing library could be established, such as parent–child education lectures and documentaries, to enhance the quality of guardianship. Conversely, it is recommended that guardians within the family establish mutual supervision and exchange information on the educational and living trends of the left-behind children through regular online group chats or phone calls, thus creating a system of care for the children within the family. Regarding health care and maintenance, since grandparents and other principal guardians are older, less receptive to new ideas, and have limited capacity and energy, parents should stay in touch and communicate with them daily, as well as make regular visits to their homes. As for the academic performance of left-behind children, migrant parents should maintain communication with teachers, seek their assistance, and promote a tolerant and encouraging environment.

### 6.3 Limitations and future directions

Due to space limitations, this paper only explores the mechanism of the influence of parenting styles on the mental health of left-behind children when their parents go out to work. In fact, depending on the guardians, the parenting styles of left-behind children can take different forms, including intergenerational parenting, kinship parenting, mother parenting, father parenting and mixed parenting. The parenting attitudes and behaviors of different guardians towards left-behind children vary to some extent, and thus the impact on their mental health is also heterogeneous. From this, we can see that there are still many different types of family guardianship for left-behind families due to their complex forms, and the characteristics of the differences in the parenting styles of different guardians and their impact on the mental health of left-behind children are still research topics that need to be further deepened and refined.

## Data Availability

The original contributions presented in the study are included in the article/supplementary material, further inquiries can be directed to the corresponding author/s.
